# Primate thanatology and hominoid mortuary archeology

**DOI:** 10.1007/s10329-019-00769-2

**Published:** 2019-10-23

**Authors:** Paul Pettitt, James R. Anderson

**Affiliations:** 1 Department of Archaeology, Durham University, Durham, UK; 2 Department of Psychology, Graduate School of Letters, Kyoto University, Kyoto, Japan

**Keywords:** Thanatology, Funerary behavior, Hominoids, Evolution, Paleolithic, Death, Corpses

## Abstract

In recent years, a thanatology of primates has become a respectable research topic, and although still sparse, observations among several taxa have shown how complex responses to the dead can be. In human evolutionary archeology, re-analysis of old ‘burial’ sites is slowly revising our view on the development of specifically human responses to the dead. We propose here the means of integrating information from the two disciplines of primatology and archeology, in support of the field of primate thanatology. We propose a terminology and a shared set of research questions, from which we generate a number of observations that can be utilized in the field, in order to establish a working dialogue and foster greater collaboration across the two disciplines.

## Introduction

In recent years, considerable advances have been made in our understanding of the behavior of an increasing number of nonhuman taxa towards dead conspecifics. In primatology, early accounts were largely anecdotal or second-hand with little verification, but researchers have come to realize the value of recording activities around and involving corpses, and as a result the field of primate thanatology is gaining both in momentum and credibility (Anderson 2011; Anderson et al. 2018; Gonçalves and Carvalho 2019). In evolutionary archaeology, while research is limited to the vagaries of archeological preservation, interest is finally turning away from old dichotomies that saw human groups that ‘buried their dead’ as ‘cognitively modern’ (whatever that is) and those that apparently did not as somehow less sophisticated, towards a more nuanced approach that recognizes that burial was relatively rare until the Late Pleistocene and that there are many ways to deal with corpses (Pettitt 2011, 2018). Long-term hypotheses for the development of mortuary behavior among the homininae are attracting attention and debate (Zilhão 2015); for the Neanderthals, re-evaluation and re-excavation of old sites has both rejected and supported previous material interpreted as burials, such as Roc de Marsal and la Chapelle-aux-Saints, respectively (Sandgathe et al. 2011; Rendu et al. 2014). Furthermore, new excavations in old sites pertinent to thanatology such as Shanidar Cave (Iraqi Kurdistan), where several Neanderthals were apparently buried, are beginning to provide a somewhat clearer picture (Pomeroy et al. 2017).

More widely, the accumulation of thanatological observations in nonhuman animals is revealing how some behaviors once thought to be ‘sophisticated’ and perhaps even exclusive to humans, are in fact widespread (McComb et al. 2006; Heinze and Walter 2010; de Waal 2013; Chouvenc et al. 2011; Renucci et al. 2011; López-Riquelme and Fanjul-Moles 2013; King 2013; Anderson 2016; de Kort et al. 2017; Bearzi et al. 2018; Gonçalves and Biro 2018; Gonçalves and Carvalho 2019). Examples include various methods of corpse disposal—described and systematically studied especially in eusocial insects and linked in particular to chemical cues—and post-mortem transport and care of dead infants—observed especially in primates and cetaceans and studied from the perspectives of strong emotional bonds between individuals as well as those species’ understanding of death. It has also been suggested that, as in early human societies, ways in which different populations of nonhuman species deal with dead conspecifics might show cultural variations (Biro et al. 2010), similar to many other cultural variations reported in primate populations (e.g., Whiten et al. 1999; McGrew 2003; Whiten 2011).

Recently, we attempted to define shared goals for an evolutionary thanatology that would encompass as inclusive a sample of animal taxa as possible, while also including modern, exclusively human sociological studies on topics such as the mortuary commemoration by humans of inorganic objects such as robots (Anderson et al. 2018). The breadth of scope of evolutionary thanatology is exemplified by a recently published themed issue of *Philosophical Transactions of the Royal Society of London B*, which included papers focusing on corpse management in eusocial insects, responses of corvids to dead conspecifics, responses to dead infants in cetaceans and primates, burials in early humans, children’s developing understanding of death, human language and mental representations of death, suicide, whether the dead have moral standing, bereavement and grief, and recent cultural transformations in human funerary practices (see Anderson et al. 2018 and accompanying articles).

Here, we propose a predominantly primatological perspective on the development of methods for a more general and hypothesis-driven thanatology of our nearest evolutionary neighbors. Modern primatology and paleoanthropology (in this sense taken to be Paleolithic and Mesolithic archaeology, i.e., that of Pleistocene and early—mid Holocene hunter-gatherers), supplemented by evolutionary psychology are, we argue, the most appropriate disciplines from which one might develop testable hypotheses about the long-term evolution of anthropoid treatment of the dead. The two are to some extent complementary; by its very nature, primate thanatology deals with the face-to-face and the here-and-now, while the archaeology of early hunter-gatherers is limited to the inorganic materials that survive, and thus it tends to relate to place, rather than to demonstrable interactions between living and dead individuals. This is not to say that place and space should not be important to non-human primate thanatology, nor of course individual interactions in past human thanatology. On the contrary, we need methodologies to tease these out of developing data. As the number of primatological case studies increases it should be feasible to explore how space and landscapes are used among non-human primates, and new discoveries and analysis of Paleolithic and Mesolithic mortuary sites—particularly in the light of modern field and laboratory techniques, should allow a nuancing of how individual identities affected mortuary and funerary behaviors.

How might the spatially focused, complex mortuary activities (or mortuary *complexes*, to use the terminology of Duday 2009) observed among human societies over the last several millennia have evolved from our primate past? How complex, and how culturally variable are the mortuary behaviors observed among present-day primates? How much ‘humanlike’ behavior and cognition can be attributed to nonhuman primates in the mortuary realm, and how might these derive from earlier, mammalian or even pre-mammalian roots? Our goals for primate thanatology are to understand how widespread, homogeneous, or variable, behaviors are, and whether these derive from chemical, emotional, rational or cultural cores. How closely does the sociology of the dead map the sociology of the living? As the nature of social interaction between living individuals becomes more complex, is it inevitable that interaction with the dead does too? Or are certain factors such as the cause of death, age at death, or agent of death responsible for the ensuing activity? What, in particular, is so special about the simple inhumations in shallow graves that define the earliest burials?

### Terminology and semantics

Fruitful cross-disciplinary collaboration and communication is best served by the common use of specific descriptors and definitions. What concepts are useful, and what language should we use? Below, we present four key behavioral categories that characterize discussions of the evolution of primate ways of dealing with the dead, in the hope that these terms might provide a useful framework for primatologists seeking to interpret their death-related observations within a wider thanatological perspective that includes modern and ancestral human mortuary activities. We make no attempt to review the available literature in depth; our intention is to provide a compromise heuristic framework for linking primatology and paleoanthropology; hopefully the project might expand to include other disciplines.

*Avoidance* reflects the deliberate avoidance of locations in which a death or deaths have occurred, or of specific places where predators (and therefore, death) are a palpable danger. In the wild, primates may temporarily avoid areas or abandon sleeping sites where conspecifics have recently been killed by predators (Altmann and Altmann 1970; Anderson 1984; Matsumoto-Oda 2015), but this might reflect fear of being attacked rather than a response to the other individual’s death per se. In one well-documented case, however, captive chimpanzees temporarily avoided the location and in particular did not sleep where a group member had recently died from natural causes (Anderson et al. 2010). Archeological evidence of avoidance could take the form of localities where hominin remains are relatively abundant but in the context of a lack of other activities, such as has been argued for the 3.1-million-year-old AL-333 *Australopithecus afarensis* accumulation site at Hadar, Ethiopia (see Pettitt 2011 for discussion).

*Corpse Interaction* is defined as any expression of strong interest in, or interaction with, a corpse (defined as *Morbidity* in Pettitt 2011). This might include inspection of the corpse for signs of life or at least some kind of reaction; e.g., looking at, probing, or blowing into the corpse’s eyes, touching or probing wounds or other body parts, and grooming. Other examples include displacing and transporting the corpse, and expressions of possible ‘compassion,’ such as care taking shown towards the corpse. Violent acts toward the corpse also come under corpse interaction, such as hitting, biting, or jumping on it, pulling out fur, nibbling parts of the corpse and other cannibalistic acts (for examples of various expressions of corpse interaction in primates see papers in this special issue, and Anderson et al. 2010; Biro et al. 2010; Cronin et al. 2011; Boesch 2012; Buhl et al. 2012; Stewart et al. 2012; Campbell et al. 2016; van Leeuwen et al. 2016; Yang et al. 2016; Pruetz et al. 2017; Porter et al. 2019).

*Mortuary activity* is a more general category that relates to any interaction with or actions stimulated by the corpse. This covers any observable behavior that can be reasonably and confidently associated with a corpse, including for example vocalizing, remaining in the vicinity of the corpse, repeatedly visiting it or the site after traces of the corpse have gone, deliberately modifying the landscape around the corpse, or interacting with others near to where the corpse is (landscape modification excepted, for examples see e.g., Teleki 1973; Boesch 2012; Pruetz et al. 2017).

*Funerary activity* is more specific, and defines any mortuary activity in which an element of active commemoration can be identified. Thus, funerary activity implies potentially higher-level cognitive processes than the other categories. In most cases, this involves simply using either the landscape (e.g., use of a particular location or topographical/landscape feature to dispose of the corpse) or tool use (“material culture”, e.g., to dig a grave, or use of vegetation or stone to mark the location of a corpse), drawing attention to its presence and thus creating an association between that place and the dead. Undoubtedly, funerary activity has evolved particularly in the human lineage (Pettitt 2011), but one aim of the present article is to encourage primatologists to give greater consideration to the possibility of some incipient forms of funerary activities around the place where they witness death of a conspecific. In many or indeed most cases, there may be adequate explanations that need no reference to commemoration. For example, Boesch (2012) reported chimpanzees in the Tai Forest dropping vegetation onto corpses that they discovered unexpectedly, but parsimoniously interpreted this as a way of investigating the corpse from a safe distance, rather than as a specifically mortuary activity. Also, individuals revisiting the location where they previously witnessed death sometimes sniff the ground and vegetation (Pruetz et al. 2017), suggesting memory for the deceased and/or the death event. Such olfactory exploration might be simply to update their information about the deceased, or the possible presence of other individuals or species (e.g., scavengers, predators), although such cases might be better subsumed under the wider context of simple mortuary behavior.

### The wider ethological and anthropological context

Until recently, the lack of detailed observations and video records of responses to their dead by primates, and the lack of detailed microsedimentological recording of archeological mortuary activity during the Paleolithic, meant that many potentially rich sources of information were not available. Modern archeological excavations benefit from highly technical forensic approaches to human death assemblages which provide considerable nuanced data on the corpse, its death, and its funerary context (Duday 2009), and post-excavation analyses have considerable analytical power to address osteoarcheological and paleopathological questions (e.g., Sandgathe et al. 2011; Sala et al. 2016; Pelletier et al. 2017; Pomeroy et al. 2017; Gómez-Olivencia et al. 2018; Sparacello et al. 2018, for recent Paleolithic examples). Molecular analysis such as isotope chemistry and ancient DNA can provide valuable biographic information on the deceased and their social context (Mittnik et al. 2016). In ideal circumstances, these combined analyses provide information akin to that extracted from both a forensic ‘crime’ scene, and a subsequent ‘autopsy’ as we might call them, even if the precise cause of death is often unknown. However, evidence for mortuary activity during the Paleolithic is scant (Pettitt 2011 and references therein), and restricted largely to burials that were recovered before modern standards of excavation, to stone tool cutmarks on human bones indicative of soft tissue removal (whether for cannibalism or more ‘ritual’ defleshing such as scalping) or fresh fractures of various forms for which ‘natural’ accidents can be discounted (see for example Sala et al. 2016), and to isolated human remains on occupation sites which may or may not derive from disturbed burials, curation of body parts (i.e., purposeful retention and carrying around of bones or teeth), or other forms of mortuary activity. Thus, most of the evidence deriving from our archeological ‘crime scenes’ is limited, even if one can deploy a suite of analyses on the human material that survives (Gowland and Knüsel 2006; Duday 2009).

In primatology, as of yet there is no archeology of mortuary activities, and maybe there never will be. Among living communities, however, the increasing number of deaths witnessed directly or discovered shortly after the event, often supplemented with detailed video records (e.g., Matsuzawa 1997; Anderson et al. 2010; Stewart et al. 2012; Cronin et al. 2011), means that more detailed qualitative and quantitative descriptions of the mortuary activities of monkeys and apes are becoming available for interpretation now, and for future reference. There are limitations, however, and in this context, we note the tendency of primate field workers, park staff, and caretakers of captive primate populations to remove a new corpse within a short period of time following the death, a practice that results in the loss of potentially useful information about subsequent primate mortuary activities. In the field, a death may rightly be looked upon as providing a source of materials for anatomical, pathological, or biochemical studies, etc., and indeed there exist recommended procedures for burial, excavation, and preparation of nonhuman primate skeletal remains (Garrod et al. 2015). Swift removal and disposal of corpses may also be justified in terms of prevention of the spread of disease (in both the field and captivity) (e.g., Porter et al. 2019), or in safari parks or zoos, to shield visitors from seeing dead animals, as the latter can give rise to negative reactions (e.g., Benbow 2004), and simply due to concerns about appropriate and ethical treatment of the dead. Such concerns are of course valid, but we call for greater consideration to be given to leaving corpses in situ whenever possible, in order to maximize information return about the responses of the living to the corpse, ideally until the group moves away and finally abandons it. The available literature strongly suggests that, except for predation and dead infant-carrying, corpses are usually abandoned within a few hours of death, so we hope that such recording need not be incompatible with the health and ethical concerns that currently determine conventional practices.

Clearly, decisions need to be made about the relative importance of obtaining more samples for morphological or biochemical research versus a better understanding of the taphonomy of primate death sites and any subsequent activities around them. Some aspects of modern forensic crime scenes could provide an appropriate model of how to proceed. One possible procedure might be to remove small samples of soft tissue, tooth and bone from a corpse in the field to be stored for DNA, isotope, and other analyses, leaving it largely intact and in the same posture and place. Such samples could be taken once the surviving members of the dead individual’s group have left the corpse and moved away from the death site, as attempts to approach a corpse can elicit intense excitement and aggressive defense of it by members of the group which would hence affect observations (e.g., Campbell et al. 2016). Like others (e.g., Watson and Matsuzawa 2018), we recommend video recording of activities in the vicinity of a corpse when feasible. Also, remote video or camera traps can record mortuary activities even in the absence of direct observation, including reactions of other species in relation to the corpse. For example, over a 25-day period, Huang et al. (2014) captured over 4000 photographs of three mammalian and one avian species scavenging on a Golden snub-nosed monkey carcass in Sichuan, China. No member of the dead monkey’s social group returned to the area, but whether this reflects active avoidance is unclear, as the preserved carcass was only returned to the site after a 3-week delay.

### Research questions for primate thanatology

With regard to extinct and extant primates including humans, mortuary activity can range from brief vocal and somatic expressions of emotional reactions to death and accompanying social displays, to the repeated use of particular places for the ritual disposal of the dead and the use of such to reflect age, gender, status, and other social differences between individuals that they presumably held in life (Binford 1971). A growing complexity of mortuary activity over time may of course reflect—or at least broadly track—cognitive evolution, although this needs to be demonstrated rather than simply assumed in progressivist evolutionary narratives. If group size and social complexity can to an extent be correlated with brain size over the course of primate evolution (Dunbar 2003), does it necessarily follow that as group complexity grows, so too does the complexity of mortuary activity, as hypothesized by Pettitt (2018)? Recent observations on primates and indeed other taxa suggest that mortuary activity can be relatively complex, although without necessarily implying any cognitively sophisticated underpinnings such as ‘symbolic’ capacities or anything that makes it specifically funerary in our sense (Anderson et al. 2010, 2018; Gonçalves and Carvalho 2019). Primate thanatology should, we suggest, focus on building up a volume of observations that can be used to test specific models about what factors promote relatively complex mortuary activities, and in particular when and why places in the landscape begin to be associated with the dead.

By focusing on our nearest living evolutionary neighbors, we can generate a core of mortuary behaviors that we might expect to have been expressed, however variably, among Miocene, Pliocene, and Pleistocene hominoids and hominins. At present, most information is available for chimpanzees, with accounts of reactions to dead conspecifics in captive, semi free-ranging, and wild settings (Anderson 2018). The most widely documented suite of responses concerns the maternal transport and caretaking of dead infants, a behavior which is not restricted to great apes (Sugiyama et al. 2009; Watson and Matsuzawa 2018; Das et al. 2018; Gonçalves and Carvalho 2019). The fact that it has been reported in multiple species and in different chimpanzee populations argues against the idea (e.g., Biro et al. 2010) that dead infant-carrying might be culturally determined, although it is possible that specific aspects of handling dead infants, or even the motivation to do it, might be socially influenced, i.e., vary between individuals, groups, or taxa; this remains to be seen. Does the apparent strength of bond between a mother and her offspring also explain the Middle Paleolithic burial of a mother with her fetus/neonate at Ostuni, Italy (Vacca and Coppola 1993), the Mid Upper Paleolithic burial of three infants at Krems-Wachtberg, Austria (Einwögerer et al. 2006, 2008), and an elderly female clasping in her arms a chondritic dwarf in the Late Upper Paleolithic levels of the Romito rockshelter, Italy (Frayer et al. 1987, 1988)? What social relationships might underpin the Mid Upper Paleolithic triple adult burials of Barma Grande in Italy and Dolní Věstonice in the Czech Republic (Formicola 1988; Formicola et al. 2001)? With Paleolithic burials—whether Neanderthals or *Homo sapiens*, are we dealing with behavior that is determined by blood relationships, or by relationships of hierarchical rank [or social status?] and competition?

More generally, questions about intra- and inter-group variability, and possible cultural influences on mortuary activity among the living primates cannot yet be answered due to insufficient observations. Going beyond dead group members other than infants, chimpanzees have variously been reported to inspect dead bodies visually and olfactorily, gently touch, caress, or hold the hand of, a deceased group member, groom the body, wave away flies and remove dirt or debris, hit, pull, jump on and drag the body, inspect and manipulate it via a stick, and in some cases to drop branches on it from above; various degrees of cannibalism have also been reported (see e.g., Teleki 1973; Anderson et al. 2010; Boesch 2012; Stewart et al. 2012; Pruetz et al. 2017). Below, we present four major questions that we believe to be both testable and pertinent for primate thanatology. The list is neither exhaustive nor in any particular order; we invite readers to prioritize the questions according to their own research circumstances, and to modify the questions as appropriate and add new ones. In Table 1, we attempt to formulate more specific questions that could guide field observations in ways that should allow us to address these major questions.

**Table 1:** Some research questions for primate thanatology and their archaeological correlates

Research question	Primatology	Archaeology
Responsive or emotional context		
Is there a particular link between infanticide, cannibalism, and fragmentation of the body? Do any individuals bite or in any way consume parts of the corpse? How many? What parts? How frequent? Does this alternate with other activities?	What percentage of dead individuals of various age- and sex classes are cannibalized, and to what extent? How do cause of death and time elapsed since death influence the likelihood of ingestion of body parts? How do patterns of consumption (e.g., order in which body parts are ingested, and which parts are not consumed) vary across populations and species of corpse (hetero- vs. conspecific)?	How frequent are isolated body parts, and do these appear in particular contexts (e.g., pits)? How frequent are cut marks, and do these reflect particular activities (e.g., nutritional cannibalism, defleshing, scalping)? How great is a concern for the redeposition of human remains disturbed by new depositions?
What are the expressions of violence towards the corpse; how variable are they, who conducts them, and how long do they persist? Are they repeated? Do they alternate with other emotional expressions, e.g., grooming?	What aggressive acts are directed towards the corpse (e.g., pounding with fists, jumping, stamping, pulling out hair, scratching, biting, tearing off flesh, etc.)? What is their time course, and how do they vary with non-aggressive corpse-directed acts?	Fresh fractures of diverse forms (e.g., crush, depression, comminuted, transverse), scrape or cutmarks on bone for which ‘natural’ accidents seem unlikely
What are the expressions of violence towards other (living) individuals in the presence of the corpse?	Does aggression feature in the interactions among those present in the vicinity of the corpse. If so, what are the details?	
Social context		
Do sudden deaths such as falls from trees and ambushes precipitate stronger/more varied/longer duration responses than deaths that end processes of decline/disease?	What are the differences in reactions to traumatic vs. more peaceful deaths? Are different kinds of traumatic deaths (e.g., predation, conspecific killing, accident) followed by different post-death reactions?	Are rarer or more elaborate forms of archeologically visible mortuary or funerary activity accorded to individuals who display signs of traumatic (i.e., violent) death?
Can one define a ‘death space’, i.e., a particular radius around the corpse, access to which is possibly restricted and within which activity is entirely corpse-focused and only without do other activities continue? What is its size, and how is this related to group size or social complexity?	In what circumstances do some individuals (e.g., dominant males) prevent others (e.g., juveniles) from approaching the corpse? How long does this last? What is the size and shape of any restricted “death zone,” and how do these vary with species, population, social, and death contexts etc.?	Do burials occur within living sites or are they emplaced in an area devoid of such? Is this pattern repeated, i.e., can one define specific areas set aside for disposal of the dead, perhaps in specific places (cemeteries)?
How long does corpse-focused activity persist? How many individuals (% of group) engage in it? Are all group members affected by the death/corpse or do some appear ‘unaffected’ by it? If not, is there any social/status/affiliation that determines who engages in mortuary activity and for how long?	Which members of the group engage in mortuary activities, for how long, and in what circumstances? What is the influence of context, place, and social status of the dead individual and the survivors present?	NA
Are calls expressed in relation to a corpse common or rare in other circumstances? Are any apparently exclusive to mortuary activity, i.e., specific responses to the corpse? If so, could this be regarded as an emergent language of death/grief? How variable are these within a specific group, i.e., how expressive are groups about death?	Are there any specific vocalizations or physical gestures elicited only by corpses? Could such behaviors, or others that occur only within the context of death reveal an awareness of death and/or grief? Again, are there individual or population differences?	NA
Does the complexity of mortuary activity, expressed as a growth in the variety of expressions/number of individuals participating/time these activities occur over and in which other activities are neglected, grow with increasing group size/complexity? To put it simply: does a more complex social group always engage in more complex mortuary behaviors?	Does the duration or complexity of mortuary activities vary as a function of group size or complexity? Might complexity of mortuary activity vary with other signs of cognitive complexity, such as tool use, or adaptations to more challenging environmental contexts (e.g., savannah vs. forest habitats, seasonally extreme vs. more stable habitats)?	Is the apparent correlation of places of multiple burial in locales set aside for the dead (‘cemeteries’) always associated with semi-sedentism or sedentism, or are they present among more mobile hunter-gatherers? Do more complex mortuary behaviors arise specifically in difficult environments such as those of the Mid Upper Paleolithic? Do these correlate with other bursts of activity such as art production?
Does the status of the deceased affect expressions of mortuary activity (e.g., do low-status individuals receive less attention than high-status individuals)?	How do reactions compare to corpses of immature vs. mature individuals, those of low- vs. high status, or those that are central vs. peripheral?	Are Paleolithic and Mesolithic burials always accompanied by relatively rich material culture? Can one distinguish between materially rich and materially poor burials at sites with multiple burials? If so, do these differ spatially? Do cemeteries from the Late Pleistocene onwards display variability of mortuary activity between individuals? If so, how? Do individuals in ‘richer’ burials show isotopic signs of distinct diet (e.g., access to more meat)? If so, does this correlate with other differences, e.g., age, gender, ‘grave goods’ or treatment of the corpse? Under what circumstances are animals (e.g., wolves, foxes) buried in ‘cemeteries’ or with humans?
Do individuals of different status respond in different ways to the corpse?	How does status or social role within the group influence responses to corpses?	NA
Material culture		
Is any material culture used to interact with the corpse? Who does this?	Do those present use objects in their interactions with the corpse? What kinds of objects, are used, and what are they used for?	Potentially any objects associated with the corpse (‘grave goods’) that cannot be explained in any other fortuitous way, such as personal ornaments on clothing &c.
Is any material culture used to affect the position or visibility of the corpse, e.g., branches to cover it, or stones to mark or surround it?	Are features of the immediate environment (e.g., vegetation, soil, rocks) manipulated in ways that affect the position or visibility of the corpse, or that might mark the location?	Are primary or secondary burials associated with grave pits/scoops, stone markers, liners or coverings, or other signs of organic materials that may be revealed through microsedimentology, palynology &c?
If any use of material culture is observed, does this vary from group to group, and/or correlate with other cultural differences?	Could any mortuary activities be added to the many known examples of cultural behaviors in nonhuman primates?	How widespread are archeological examples of material cultural association with mortuary evidence, and do they vary diachronically and/or spatially?
Space and landscape		
Is the corpse transported? If so, by whom, for how long, where to, how far? Is this a singular or repeated activity? Does it appear to be deliberate or can one not rule out random abandonment?	If attempts are made to move or drag the corpse, who does this, how frequently, and over what distance? When the corpse is eventually abandoned, does the place of abandonment appear to be chosen deliberately, or is it random?	How intact or fragmentary are human remains in mortuary or other contexts? Does this, and their preservational state, suggest a complex pattern of fragmentation and curation?
Does the group avoid locations in which predators are active and death is, therefore, a danger?	Can active avoidance of places where death occurred be distinguished from normal focusing of ranging and foraging in other parts of the home range?	Is archeology rare or absent from major carnivore accumulation sites?
Where or in what contexts can we see examples of space or place becoming incorporated into mortuary activity?	Repeated use of a locale for mortuary activity, and particularly evidence of funerary activity	Are the dead associated with particular places, and is this associated apparently repeated?
Is it just the corpse that is the focus of activity, or does its spatial position seem important (e.g., its location or associations?)	Observations of spatial dynamics during mortuary activities	Can one identify particular areas of a site (e.g., the rear of caves or rockshelters or areas away from the living)?
If the corpse is disarticulated, are body parts removed from the corpse, and if so, curated more widely?	Other than mothers continuing to carry partially decomposed or mummified remains of their infants, are their examples of removal and possession of body parts?	Where corpses are incomplete, what parts are missing, and can these be explained taphonomically, without recourse to more complex explanations (e.g., does the schlepp effect account for missing phalanges &c)?
Does a death and the ensuing mortuary activity affect ranging behavior? For how long does a group’s activity remain focused entirely or in the main on the corpse rather than foraging?	What are the consequences of a death for the group’s daily activity profiles, including ranging, foraging, and socializing? How long do any changes last?	NA
Do primates ever return to death sites? Can they become incorporated into a foraging round?	Is there any evidence of a death site being visited more frequently than expected based on the group’s normal ranging patterns?	NA

1. Does the complexity of mortuary activity among primates increase with increasing group size or complexity (e.g., including increasing evidence of theory of mind, long-term social relationships based on kinship and friendship, exchange of social goods or services), and might this provide a mechanism or reason for mortuary evolution among the hominins? The Paleolithic record certainly indicates that mortuary behavior is more evident and more variable from the Late Middle Pleistocene and early Upper Pleistocene, among Neanderthal and *Homo sapiens* groups with encephalization quotients demonstrably higher than their Middle Pleistocene predecessors. But does this development pertain only to these groups, or is it a more general rule?
2. Do ‘bad’ deaths, those that are sudden or unpredicted, occasion more interest and activity than those that seem more ‘expected’? This may pertain in particular to infants or adults in their prime. Pettitt (2018) hypothesized that this is the case for chimpanzees; in six recorded examples more individuals engaged in corpse-related behaviors such as corpse interaction and social displays, and for longer periods of time, in situations where deaths resulted from tree falls or predator ambush, i.e., were unexpected and traumatic. But the observations are few and incomplete: what percentage of the total group was distracted from other activities by these deaths; do responses across populations vary in ways that suggest different cultures? Mid Upper Paleolithic burials are often of individuals with observable pathologies and/or violent or otherwise sudden deaths (Trinkaus et al. 2001; Formicola 2007). An ethnographic survey of diverse hunter-gatherer groups showed that they tend to believe that death is natural (i.e., inevitably comes to everyone) except in the case of the very young and adults in their prime (Binford 2004). The universality component of the death concept (i.e., the acknowledgement that everyone dies) is an open question for primatology, especially in the great apes, given their capacities for self-awareness (Anderson and Gallup 2011, 2015).
3. How widespread is the concern to cover or otherwise hide a corpse, and does this relate to the processes of necroclaustralization (covering) and necrophoresis (removal) observed among various insect taxa? The relative paucity of burials before the rise of semi-sedentary, complex hunter-gatherers of the Late Pleistocene suggests that simple abandonment or necrophoresis may have pertained for much of the course of human evolution. A major watershed would therefore be the rise of the practice of funerary caching—the deliberate deposition of corpses in natural places such as fissures and caves—among *Homo heidelbergensis* groups from the mid Middle Pleistocene (~ 500,000 BP), and *Homo neanderthalensis* and *Homo sapiens* in the late Middle Pleistocene and Upper Pleistocene ~ 115,000 BP onwards (Pettitt 2018). This suggests that by this time hominins had extended the process of corpse removal from campsites to their deliberate deposition at specific places. Thus, can we recognize an evolutionary development from abandonment to necrophoresis to necroclaustralization, ultimately resulting in specific places for the disposal of the dead? With the possible exception of dropping vegetation on “unexplained” conspecific corpses by chimpanzees (Boesch 2012), there is as yet little evidence of corpse caching in nonhuman primates. Nor has moving or attempting to move corpses to *specific locations* been recorded, but we believe that it would be worthwhile to pay greater attention to recording distances over which corpses are sometimes dragged, and the precise types of location where they are eventually abandoned. Other pertinent questions include, for example, whether larger primate groups take longer to fully abandon a corpse. Might attempts to move the corpse be related to the size and social status of the individual when alive, and aspects of the social group such as age-to-sex ratio, absolute size etc.?
4. Is there a general rule that the stronger the social attachment between individuals, the stronger or more protracted the process of *detachment*, (sensu Gamble 1999); i.e., expressions of corpse interaction, grief, mortuary, and funerary activity? This question in relation to nonhuman primates has been addressed with particular references to mothers’ responses to their dead infants (Sugiyama et al., 2009; Anderson 2017; Watson and Matsuzawa 2018; Das et al. 2018; Gonçalves and Carvalho 2019), but recent reports have highlighted notable behaviors by individuals towards the corpses of non-kin individuals with whom they shared strong social bonds before the death (Anderson et al. 2010; Bezerra et al. 2014; van Leeuwen et al. 2016; Yang et al. 2016). Closer analyses of post-death behaviors in relation to pre-death social relationships in extant primates appears fundamental for the development of primate thanatology.

To clarify how to approach these major questions, we divide primate thanatology into several heuristic areas, namely: responsive (or emotional) context, social context, material culture, space, and landscape. We do not consider our research questions or heuristic areas to be in any way exhaustive, and we are aware of the speculative nature of some of them; but they can hopefully serve as a way of orienting primatologists towards the kinds of observations that would help address a wide range of hypotheses about the long-term evolution of primate—particularly great ape—mortuary activity. We similarly want to orientate archeologists to a meaningful discourse with primatologists. Such a discourse can address the issue of what constitutes the major changes from an ape-like to a human-like mortuary behavior, one that extends from the ‘face to face’ to place. What factors determine how the temporal or spatial scale of such behaviors increases within and between groups and over the course of hominoid evolution? Is mortuary activity usually more focused on the dead (e.g., their status in life, ties to the living, or cause of death) or on the living (e.g., how individuals renegotiate their position in the group social structure after a death); are there any vocalizations, postures, or behavioral displays (e.g., unusual calls, copulation, aggression or other behaviors) that specifically address the corpse, the living, or both?

Table 1 presents some specific research questions and observation desiderata for primatology and archaeology, organized according to the main heuristic contexts outlined above. Rather than being complete or containing questions to be equally addressed by both disciplines, we see this list as a guide to action, providing food for thought in the field, and a proposal to share objectives using a unified terminology. Existing reports pertinent to some of questions for primatology are cited in Anderson (2017, 2018), Gonçalves and Biro (2018), Gonçalves and Carvalho (2019), and Watson and Matsuzawa (2018); for archeology see Pettitt (2011, 2018) and Zilhão (2015).

## Conclusions

Our proposals here are a first, and therefore a modest attempt to begin developing a shared terminology and methodology between primatologists and archeologists. We argue that archeologists and primatologists are particularly well suited to undertaking this together. The extent to which observations on the living in the present world can be meaningfully linked to analyses of excavated materials pertaining to the long-dead is of course questionable, and will leave much to be desired. But we believe that there is enough scope for at least a cautious joint project on the long-term development and diversity of mortuary behaviors. The project is relevant to primatologists and other animal behavior researchers, psychologists, anthropologists, and archeologists, among others. The field is new, and observations still scant. For this reason, we believe it important to develop an observational methodology now, with which further observations can be documented in ways as to maximize their utility. We may be sure that a number of our proposed questions will remain unanswered or unanswerable; that our methodologies will remain in need of further refinement and correction; and that there will be alternative perspectives on primate thanatology. But we strongly feel that it is a project worth pursuing.
